# Grouping and Crowding Affect Target Appearance over Different Spatial Scales

**DOI:** 10.1371/journal.pone.0071188

**Published:** 2013-08-13

**Authors:** Bilge Sayim, Patrick Cavanagh

**Affiliations:** 1 Laboratoire Psychologie de la Perception, Université Paris Descartes, Sorbonne Paris Cité, Paris, France; 2 CNRS UMR 8158, Paris, France; 3 Laboratory of Experimental Psychology, University of Leuven, Leuven, Belgium; Nothwestern University, United States of America

## Abstract

Crowding is the impairment of peripheral target perception by nearby flankers. A number of recent studies have shown that crowding shares many features with grouping. Here, we investigate whether effects of crowding and grouping on target perception are related by asking whether they operate over the same spatial scale. A target letter T had two sets of flanking Ts of varying orientations. The first set was presented close to the target, yielding strong crowding. The second set was either close enough to cause crowding on their own or too far to cause crowding on their own. The Ts of the second set had the same orientation that either matched the target’s orientation (Grouped condition) or not (Ungrouped condition). In Experiment 1, the Grouped flankers reduced crowding independently of their distance from the target, suggesting that grouping operated over larger distances than crowding. In Experiments 2 and 3 we found that grouping did not affect sensitivity but produced a strong bias to report that the grouped orientation was present at the target location whether or not it was. Finally, we investigated whether this bias was a response or perceptual bias, rejecting the former in favor of a perceptual grouping explanation. We suggest that the effect of grouping is to assimilate the target to the identity of surrounding flankers when they are all the same, and that this shape assimilation effect differs in its spatial scale from the integration effect of crowding.

## Introduction

**Figure 1:**
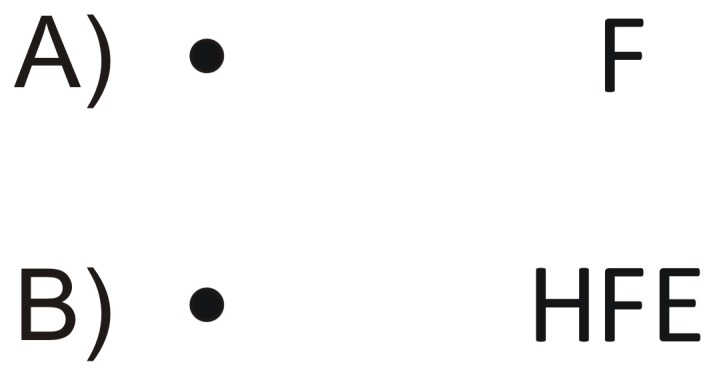
Demonstration of crowding. A) When fixating on the disc, most observers easily identify the target letter F in the periphery. B) When the target is flanked by other letters, identification is compromised.

Target perception in the periphery is impaired by nearby flankers, an interference effect that is called crowding, e.g., [1]–[3], see also [4]; for recent reviews see [5]–[7]. For example, identification of a target letter is compromised when it is flanked by other letters (Figure 1; [3], [8]–[10]). Crowding has been explained by several - not necessarily mutually exclusive - mechanisms, such as pooling [11]–[12], excessive feature integration, e.g., [10], substitution [13]–[14] and limits of attentional resolution [15]–[16].

Most accounts of crowding agree that crowding is a form of integration over space: target features are spuriously combined with flanker features, e.g., [10]–[12], but see [14]. In fact, the spacing between the target and the flankers is one of the most important factors that determine crowding – close-by flankers yield stronger crowding than flankers that are farther away, e.g., [8]. It is often proposed that the region in which flankers cause crowding, the critical spacing, is around 0.5 times the eccentricity of the target in radial direction (also referred to as Bouma’s law; [3]) and about half that size in tangential direction [8]. However, crowding strongly depends on the similarity between the target and the flankers [17]–[22]. For example, several studies have shown that crowding is reduced when the target differs from the flankers in basic features, such as color [18], [21], [23]–[24], see also [20], contrast polarity [18], or orientation [2], [25]–[27].

**Figure 2:**
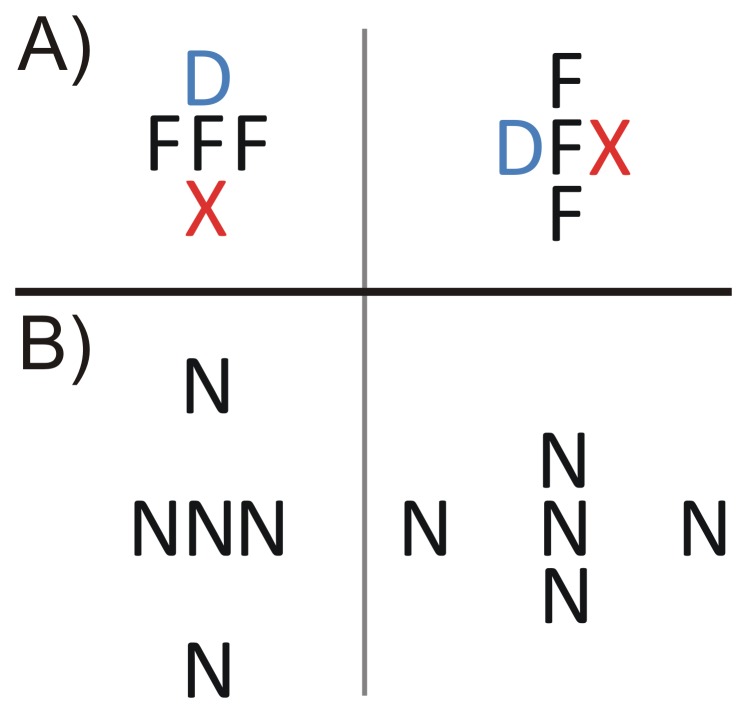
Demonstration of grouping. A) The central letter F groups more strongly with the other Fs than with the D and the X – grouping depends on the similarity between items. B) The central letter N groups more strongly with the two close-by Ns than with the far Ns – grouping depends on the spacing between items.

All these characteristics of crowding – integration over space, dependence on spacing and dependence on similarity – bear striking similarities to visual grouping. Visual grouping is also a form of integration over space, namely, separate elements of an image are seen to “belong together”, e.g., [28]–[30]. Furthermore, as depicted in Figure 2, smaller spacing and higher similarity are associated with stronger grouping (“law of proximity” and “law of similarity”).

Relations between crowding and grouping have been reported in a number of crowding studies [24], [31]–[41]. For example, it has been proposed that the degree to which a target groups with the flankers determines crowding: the more the target groups with the flankers the stronger the crowding [24], [36]–[41]. The prominent role of target-flanker grouping is also apparent in studies that showed correlations between separate measures of crowding and grouping. For example, crowding strength is negatively correlated with subjective judgments of the “standing out” of the target from the flankers [39]. The more a target was judged to stand out from the flankers (i.e., to “ungroup” from the flankers), the weaker the crowding. Importantly, these studies suggest that the properties of similarity and proximity that are important for crowding may be just a subset of the many factors that determine how well the flankers group with the target and, as a consequence, how strongly they crowd the target.

While grouping and crowding are therefore clearly interrelated, we still do not know the mechanisms underlying grouping and crowding. The classical phenomenon of grouping is, at first glance, quite unlike crowding. Specifically, when elements group together, they appear to be connected as if they belong together. In contrast, when flankers crowd a target, the target does not just appear to belong with the flankers, but additionally its appearance is altered. Its identity is lost as its features are combined with those of the flankers. If this is grouping, it is of an extreme sort. Nevertheless, under some circumstances, grouping is reported to change the perceived shapes of the individual elements so that they not only appear to belong together, they also appear more similar [42]–[43], see also [44]–[45]. It is this type of grouping that interests us here. We ask whether the change of appearance by grouping is the same as that observed in crowding by investigating the spatial scale of grouping and crowding. We hypothesize that the influence of grouping on the appearance of a target operates over a larger spatial extent than that of crowding.

In our experiments, we do show that grouping with elements *outside* the crowding radius can affect target appearance. Hence, effects of crowding and grouping on target perception are subject to different spatial constraints, and are therefore distinct, in particular in regard to their influence on target appearance. In contrast, if crowding and grouping effects on target appearance were identical, flankers outside the critical spacing of crowding should not affect target identification irrespective of whether the flankers were matched to the target (favoring grouping) or not.

**Figure 3:**
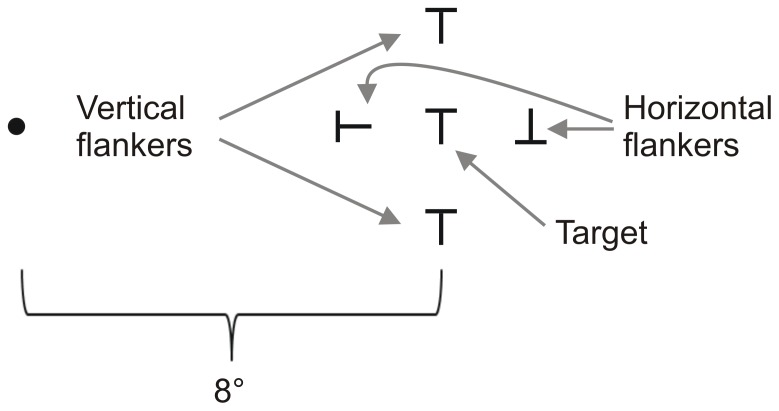
The stimulus consisted of 5 oriented Ts, presented at 8°, randomly to the left or right of a central fixation dot (indicated by the black disc on the left). Observers indicated the orientation of the T in the center (0°, 90°, 180°, or 270°). The target was flanked by two sets of flankers: “Vertical” flankers and “Horizontal” flankers.

In three experiments, we presented a target letter T of varying orientations flanked by two sets of Ts (flankers), making a cross-shape with the target in the center (Figure 3). One subset – we call Horizontal flankers – was always presented inside the critical spacing and assured that the target was strongly crowded. The other subset – we call Vertical flankers – was presented either inside or outside the critical spacing.

In Experiment 1, the Vertical flankers had, on some trials, the same orientation as the target (Grouped condition) and on others, different orientation (Ungrouped condition). We found better performance when the target had the same orientation as the Vertical flankers, i.e., when the target grouped with the Vertical flankers, compared to a different orientation, i.e., when the target did not group with the Vertical flankers. This was equally true when the matched flankers were inside as when they were outside the critical spacing for crowding.

In Experiment 2 using a Yes/No task, we found that the effect of grouping from the Vertical flankers was to change bias not sensitivity. Specifically, when the Vertical flankers both had the same orientation, subjects were more likely to respond as if that orientation was what they saw at the target location, no matter which orientation was present there. In Experiment 3A, to expand this finding, we used a larger number of Vertical flankers, modified the measurement of the critical spacing, and tested a larger set of distances between the target and the Vertical flankers. Results again showed that grouping affected bias not sensitivity.

Was this bias effect from the Vertical flankers just a response bias? Given the difficulty in seeing the actual target, the salience of the Vertical flankers, when they all had the same orientation, may have directly biased the response. Alternatively, the grouped flankers may have had a perceptual effect: filling in the target identity when it was degraded by the crowding from the Horizontal flankers. This change in perceived appearance has been previously reported for grouped elements under other circumstances [42]–[43], see also [44]–[45]. In Experiment 3B, we examined whether the bias arose at the perceptual or response stage. To do so, we varied the horizontal position of the Vertical flankers with the rationale that a perceptual grouping effect should only occur when the Vertical flankers were aligned with the target. In contrast, response bias should persist as long as the Vertical flankers remained salient. The bias was only observed with the Vertical flankers horizontally aligned (or nearly aligned) with the target. This result favored a perceptual bias over a response bias.

Our results suggest that grouping changes appearance by assimilating signals to the common identity of the group and that this appearance change is most evident for elements that are weakened and already difficult to identify, in our case, because they were crowded by other flankers. Our results show that the effects of grouping and crowding on target appearance act over different spatial scales, and hence, that in this regard crowding is not the same as grouping.

## Experiment 1: Orientation Discrimination

### Materials and methods

#### Observers

Five experienced psychophysical observers, including one of the authors, participated in the experiment (two females, three males). Four observers were naive as to the purpose of the experiment. All observers had normal or corrected-to-normal visual acuity.

All experiments were carried out according to ethical standards specified in the Declaration of Helsinki and were approved by the Ethics Committee from the Université Paris Descartes. As authorized by the ethics approval, consent for this psychophysical procedure was verbal. All recruited participants gave informed consent as documented by the data records.

#### Apparatus

Stimuli were presented on a 22″ Formac ProNitron 22800 CRT monitor driven by a standard accelerated graphics card. The screen resolution of the CRT was set to 1056 by 792 pixels. Observers were supported by a chin and head rest and viewed the monitor from a distance of 65 cm. The experimental room was dimly illuminated. Responses were recorded using a standard keyboard. MATLAB 7.5 (Mathworks, Natick Massachusetts, USA) in combination with the Psychophysics toolbox [46] was used for stimulus presentation and data collection.

#### Stimuli

Stimuli consisted of capital letters T presented at a horizontal distance of 8 degrees to the left or right of fixation. The target T was presented in the center of two sets of flankers (Figure 3). The first set – “Horizontal flankers” – consisted of two Ts of varying orientations (upright, or rotated 90, 180, or 270 degrees from the upright position, but never of the same orientation as each other and as the target) placed 2.0 degrees to the left and right side of the target (the innermost flanker at 6.0 degrees, the outermost flanker at 10.0 degrees from fixation). The second set – “Vertical flankers” – consisted of two Ts both with the same orientation placed above and below the target. There were two distance conditions: In the first condition, the Vertical flankers were placed near the target, in the second condition they were placed far from the target (spacing was individually adjusted, see “Design and Procedure”).

All characters were white with a luminance of 26.0 cd/m^2^ and were presented on a gray background (9.5 cd/m^2^). The two bars making up the Ts were 0.5 degrees long and 0.06 degrees wide. Each T was presented either upright, or rotated 90, 180, or 270 degrees from the upright position. A white fixation dot (26.0 cd/m^2^) was presented in the center of the screen.

### Design and Procedure

Observers fixated on the dot in the center of the screen. After 800 ms the stimulus was presented either to the left or right of fixation for 150 ms. After stimulus offset, the fixation dot was presented until the next trial. The next trial started 800 ms after the observers’ response. Observers indicated the target orientation by pressing one of four buttons. Presentations to the left and right side of fixation were counterbalanced and stimuli were presented not more than four times in a row to either side.

Before the main experiment, the critical spacing for the Vertical flankers was determined for each observer individually with the following procedure. The Vertical flankers were placed at 5 different distances below and above the target (1.2°, 1.8°, 2.4°, 3.0°, and 3.6° center-to-center distance). The Horizontal flankers were not presented. Within a trial, the orientations of the two flankers were random with the constraint that they neither had the same orientation as each other, nor the same orientation as the target. Note that for this measurement of the critical spacing the flankers never matched the target. Observers performed two blocks of 100 trials. Target-flanker distances were counterbalanced and pseudorandomly intermixed within a block. Psychometric functions were fitted to the data using the psignifit toolbox for Matlab (see http://bootstrap-software.org/psignifit/) which implements the maximum-likelihood method described by Wichmann and Hill [47]. Target-flanker distance values near to the target (at 40% correct responses) and far from the target (at 90% correct responses) were extracted individually for each observer (chance performance was 25% correct responses). These distances were used for two experimental conditions in the main experiment, in the following called “Near” and “Far”. The distances between the target and the Vertical flankers for the five observers were 1.7°, two times 1.9°, and two times 2.1° (averaging 1.9°) in the Near condition and 2.0°, 2.4°, two times 2.7°, and 3.3° (averaging 2.6°) in the Far condition (center-to-center distance). Note that the Vertical Flankers in the Far condition were presented at about 0.33 times the eccentricity, i.e. well above estimates of the critical spacing of tangential flankers [8].

In the main experiment, targets were flanked by the Vertical flankers placed at the individually determined distances (Near and Far) and by the Horizontal flankers at a distance of 2.0° to the left and the right of the target. Target and Vertical flankers had the same orientation in 25% of the trials; this is the “Grouped” condition. In 75% of the trials, target and Vertical flankers had different orientations; this is the “Ungrouped” condition. Note that the Vertical flanker orientation was not informative about the orientation of the target. In the baseline condition, only Horizontal flankers and no Vertical flankers were presented. Unflanked targets were easily discriminated by all observers. In each condition (Near, Far, and baseline), observers completed 2 blocks with 80 trials per block. Grouped and Ungrouped conditions were randomly intermixed within a block. The Near and Far conditions were measured in separate blocks.

To verify our grouping manipulation, five additional observers, who had not participated in the main experiment, rated the grouping strength of the Vertical flankers (including the target) on a scale from 1 (very little grouping) to 7 (very strong grouping) using samples of the original Grouped and Ungrouped stimuli in the Near (flanker spacing 1.9°) and Far (flanker spacing 2.6°) conditions. Each configuration was presented at the same eccentricity and size as in the main Experiment and remained until the rating was given, then the next configuration was presented, in a random order. Observers were instructed to maintain fixation throughout. Configurations from all four conditions (Grouped/Ungrouped and Near/Far) were presented 2 times to each observer and the ratings were averaged. Ratings of grouping were significantly stronger for the Grouped (mean = 5.6) compared to Ungrouped (mean = 3.7) stimuli in the Near condition (t(4) = 7.757, p = 0.001), and in the Far condition (4.3 vs 3.0, t(4) = 6.5, p<0.01).

### Results

**Figure 4:**
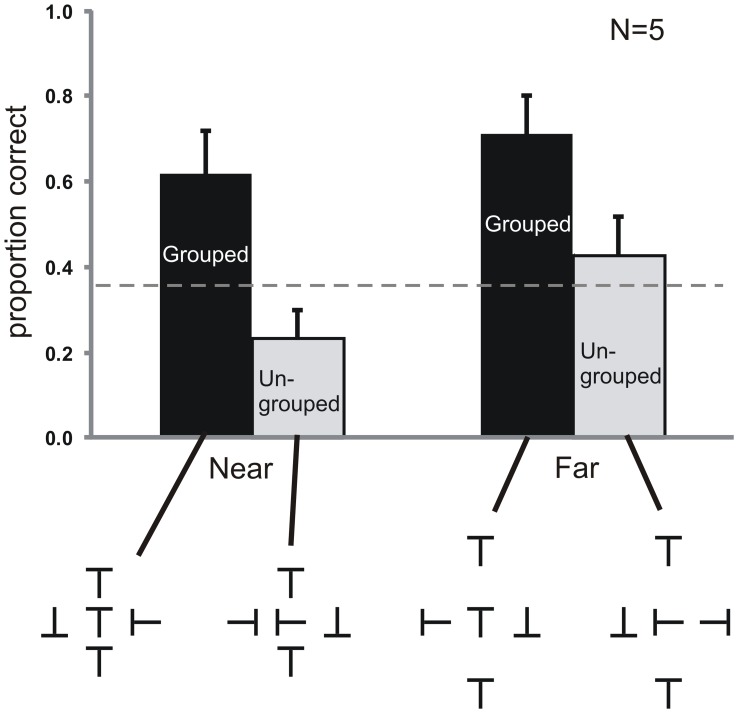
Results of Experiment 1. Performance in the Grouped conditions was better than in the Ungrouped conditions. The spacing of the Vertical flankers had no effect when they were Grouped implying that the grouping effect was unaffected by distance. However, when the Vertical flankers were ungrouped, they degraded performance when they were near, showing an ordinary crowding effect. Here, only the condition with upright Ts is shown, but all orientations were tested. The dashed line indicates performance when only Horizontal flankers were presented (baseline). Chance performance is 0.25. Error bars indicate standard errors of the mean. Stimuli are not drawn to scale.

Figure 4 shows the results of Experiment 1. The data was analyzed with a repeated measures ANOVA with the two factors Grouping (Grouped and Ungrouped condition) and Spacing (Near and Far condition).

Proportion correct was higher in the Grouped compared to the Ungrouped condition (main effect of Grouping, F(1,4) = 39.99, p<0.005). Planned contrasts revealed that the proportion correct was higher with Grouped flankers (0.62) compared to Ungrouped flankers (0.23) in the Near condition (F(1,16) = 21.51, p<0.001) and in the Far condition (Grouped: 0.71; Ungrouped: 0.43; F(1,16) = 11.52, p<0.005). Hence, performance was better when the target had the same orientation as the Vertical flankers compared to when it had a different orientation.

We also found a main effect of spacing with better performance in the Far compared to the Near condition (F(1,4) = 16.47, p<0.05). A planned comparison between the two Grouped conditions showed that there was no difference between the Near and Far condition (F(1,16) = 1.21, p = 0.29). Comparing the two Ungrouped conditions (Near and Far) revealed better performance in the Far compared to the Near condition (F(1,16) = 5.49, p<0.05). Hence, the Ungrouped (but not the Grouped) Vertical flankers reduced performance in the Near compared to the Far condition implying crowding by the Vertical flankers, as would be expected at this distance. There was no interaction between the two factors (F(1,4) = 2.46, p = 0.19). Overall, the individual data showed a similar pattern across observers with variance coming largely from a shift in mean performance from observer to observer.

Finally, beyond the analysis of proportion correct, we also looked for bias effects by comparing responses (up, down, left, and right) as a function of the orientation of the Vertical flankers – irrespective of the actual target orientation. Observers reported the target orientation of the Vertical flankers on 50% of the trials in the Near condition and 41% in the Far condition, much more frequently than the 25% that would be expected if the responses were unbiased. These indications of bias driven by the Vertical flankers raise the question of whether the better performance in the Grouped conditions compared to the Ungrouped conditions is driven by sensitivity or bias differences. We address this question in the next experiments.

## Experiment 2: Sensitivity and Bias (Yes/No Task)

The improvement in performance with the Grouped flankers in Experiment 1 may reflect enhanced discrimination of the target when it groups with the Vertical flankers and thereby ungroups from the Horizontal flankers that strongly crowd the target. Alternatively, the results may indicate a bias towards reporting the Vertical flanker orientation because it is the most frequent orientation present in the stimulus, possibly standing out from the differently oriented items in the display (in Experiment 3 we address the nature of any potential bias). In this experiment, we used a Yes/No task to investigate the effects of the Vertical flankers separately on sensitivity and bias.

### Materials and Methods

Observers and apparatus were the same as in Experiment 1. Stimuli and procedure were the same as in Experiment 1 except the following. A single response orientation was randomly assigned to each observer per block. Half of the trials contained the response orientation. Observers indicated by keyboard press whether the target at the center of the array matched the predefined response orientation, responding present or absent.

There were two flanker conditions, each comprising 50% of the trials. In the Matched condition, the Vertical flankers matched the response orientation. In the Unmatched condition, the Vertical flankers did not match the response orientation (details see below). In each condition, the central target had the predefined orientation in half of the trials (requiring a “present” response). When the predefined target orientation was presented in the Matched condition, all three vertically arranged Ts had the same orientation, corresponding to the Grouped condition of Experiment 1; when a different orientation was presented, the two Vertical flankers matched each other but not the central target, corresponding to the Ungrouped condition of Experiment 1 (requiring an “absent” response).

In the Unmatched condition, the Vertical flankers did not match each other or the predefined response orientation. For each observer, a single pair of Vertical flanker orientations was used in each, the Matched and the Unmatched conditions per block. For example, for one observer Matched flanker orientations were 270 degrees and Unmatched flanker orientations were 0 degrees for the upper flanker and 90 degrees for the lower flanker with a response orientation of 270 degrees. As in Experiment 1, the orientations of the two Horizontal flankers were randomly varied with the constraint of neither having the same orientation as each other nor the same orientation as the target – so they occasionally matched one of the Vertical flankers in the Unmatched condition, however, never the response orientation. In contrast to Experiment 1, the method of Experiment 2 ensured that a potential bias to report the Vertical flanker orientation would not result in an increase in performance, now measured by sensitivity. For example, if participants always indicated target present when the Vertical flankers matched the target orientation, half of the responses would be correct and the other half wrong (i.e., sensitivity = 0). The orientation of the Vertical flankers was not informative in regard to the presence or absence of the predefined target orientation at the center. Observers performed 2 blocks of 120 trials.

Again, we collected subjective ratings of grouping strength for the Vertical flankers using the same 5 observers and procedure as in the subjective ratings of Experiment 1. Half of the Matched stimuli contained the target, i.e. the target had the same orientation as the Vertical flankers, the other half did not. Grouping strength was higher for Matched than for Unmatched stimuli in both the Near condition (4.9 vs 3.2, t(4) = 3.9, p<0.05) and the Far condition (4.6 vs 2.8, t(4) = 4.431, p<0.05).

### Results

**Figure 5:**
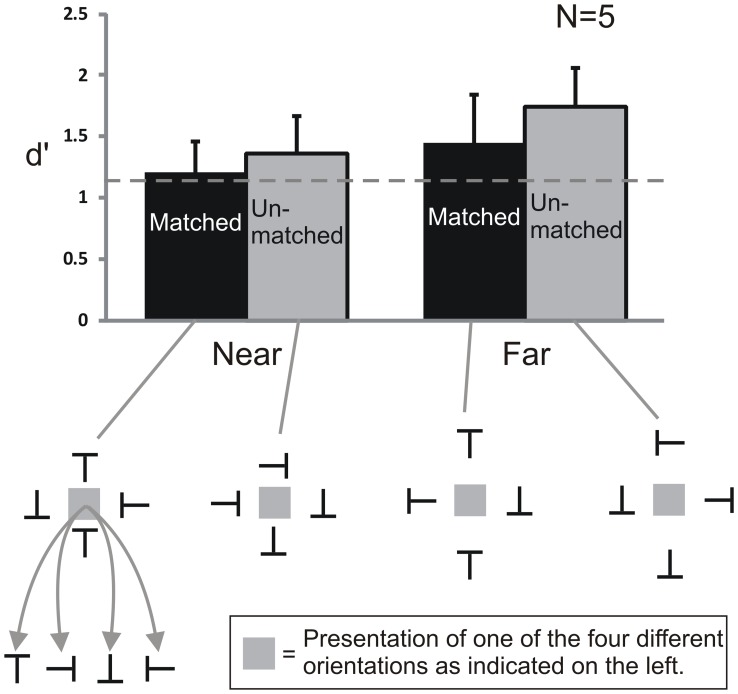
Results of Experiment 2, sensitivity. In the Near condition, no difference in sensitivity (d′) between the Matched and Unmatched condition was found. Also in the Far condition there was no difference between the Matched and Unmatched condition. Gray squares are placeholders for the target letters of varying orientations; the squares were not presented. The dashed line indicates performance on a target presented with only Horizontal flankers. Error bars indicate standard errors of the mean. Stimuli are not drawn to scale.

**Figure 6:**
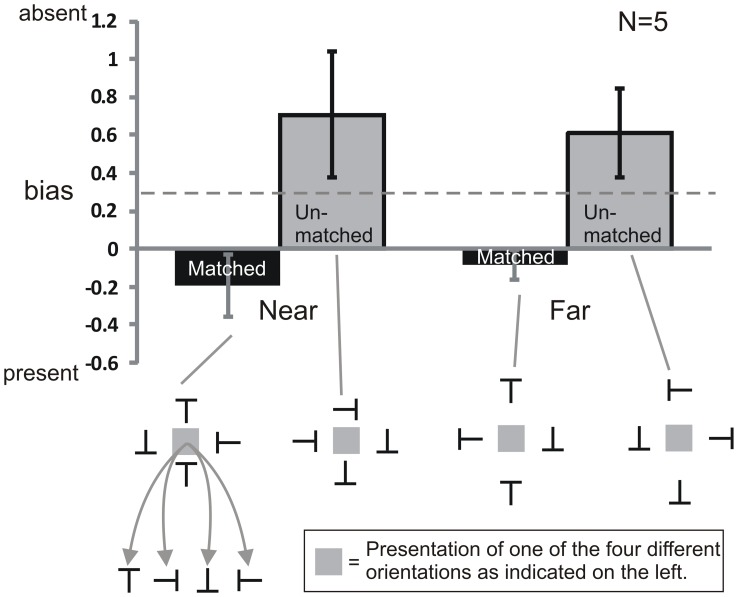
Results of Experiment 2, bias. In the Near condition, there was a difference between the biases in the Matched and the Unmatched condition. Also in the Far condition, there was a difference between the Matched and Unmatched condition. Gray squares are placeholders for the target letters of varying orientations; the squares were not presented. The dashed line indicates performance on a target presented with only Horizontal flankers. Error bars indicate standard errors of the mean. Stimuli are not drawn to scale.

The results of Experiment 2 are shown in Figures 5 and 6. The data was analyzed with repeated measures ANOVAs with the two factors Matching (Matched and Unmatched) and Spacing (Near and Far condition). Separate ANOVAs were conducted for the dependent variables d′ and bias. D′ was calculated using the standard equation
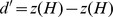
where z(H) is the z transformation of the hit rate and z(F) the z transformation of the false-alarm rate. The bias was calculated using the bias measure c (criterion) with the equation
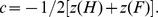
(For a discussion of different bias statistics, see, e.g. [48].)

Figure 5 shows sensitivity (d′) results. There was neither a main effect of Matching (F(1,4) = 4.816, p = 0.093) nor of Spacing (F(1,4) = 4.849, p = 0.092) and no interaction (F(1,4) = 0.098, p = 0.769). Hence, there was neither a difference between Matched (d′ = 1.21) and Unmatched (d′ = 1.36) in the Near condition, nor in the Far condition (Matched d′ = 1.45; Unmatched d′ = 1.74). In other words, sensitivity did not improve when the Vertical flankers matched each other and the response orientation compared to when they did not match. Overall, sensitivity was low in all conditions, i.e. targets were strongly crowded, also in the baseline condition where no Vertical flankers were presented (d′ = 1.18). This strong crowding by the Horizontal flankers alone possibly caused a floor effect, and hence the absence of a difference in d′ between the Near and the Far condition.

Figure 6 shows bias results. We found a main effect of Matching (F(1,4) = 8.167, p<0.05), no main effect of Spacing (F(1,4) = 0.003, p = 0.96) and no interaction (F(1,4) = 0.726, p = 0.442). A planned contrast revealed that in the Near condition, Matched flankers yielded a smaller (negative) bias (−0.2) than Unmatched flankers (bias = 0.71; F(1,16) = 8.73, p<0.01). Also in the Far condition, a planned contrast showed that there was a difference between the Matched (bias = −0.08) and the Unmatched condition (bias = 0.61; F(1,16) = 5.24, p<0.05).

A positive bias is expected when a target is crowded because an observer who cannot identify the target is likely to respond “target absent”. So in the baseline, standard crowding configuration where no Vertical flankers were present, the bias was positive (0.32). The effect from the grouping of the Vertical flankers, however, was a strong bias to report that the target was present whether or not it was.

## Experiment 3: Response Bias

These substantial bias effects could be response bias: when there are a lot of Ts of the same orientation around, observers may have a bias to report that the target is also that same orientation, in particular, when the target is so degraded by crowding that no identifying features can be seen. Alternatively, the effect of the Vertical flankers when they all have the same orientation might be to impose a common percept on the degraded target, a form of filling in or assimilation. This effect is seen with noise letters in a word context [49] and with noise targets in a crowding context [50]. The purpose of this third experiment was to distinguish between response and perceptual bias explanations. To do so, we shifted the alignment of the Vertical flankers away from the target. This decreases perceptual grouping but does not affect the frequency and salience of these flankers – it should therefore reduce any perceptual bias from grouping but not affect any response bias. We first extended the configuration of Experiment 2 to have more power to detect the effects of grouping and then we misaligned the Vertical flankers to manipulate the grouping strength without changing the salience of the flankers (which we assume leads to response bias). The results show that the bias effect is only seen when the Vertical flankers are presented at the same horizontal position as the target or close to it. These results argue against a response bias as the source of the bias from the Vertical flankers when they are all the same.

### Materials and Methods

In Experiment 3A, four of the observers of Experiments 1 and 2 (including one of the authors) and three new, experienced psychophysical observer participated (4 females, 3 males). In Experiment 3B, five of the observers of Experiment 3A (2 females) participated, all naïve to the purpose of the experiment.

Experiment 3A was the same as Experiment 2 with the following changes. Instead of two Vertical flankers, four Vertical flankers – two above and two below the target – were presented to increase the effect of grouping. In the Matched condition, all four Vertical flankers had the same orientation as the predefined response orientation. In the Unmatched condition, the two Vertical flankers neighboring the target differed in orientation from each other and from the predefined response orientation. Each of the two outermost Vertical flankers had either the remaining fourth orientation or the orientation of the Vertical flanker on the opposite side next to the target. As in Experiment 2, the target appeared half the time as the response orientation, and half the time as one of the other three orientations (chosen randomly) so the flankers themselves were not predictive of the response. The distance between the target and the Vertical flankers was varied (five conditions: 1.0°, 1.5°, 2.0°, 2.5°, and 3.0°, center-to-center distance between each two vertically neighboring Ts). The spacing conditions were presented in random order in separate blocks. For each spacing, observers performed 2 blocks (the second time in opposite order) with 120 trials each.

Using the same 5 observers and procedure as in the subjective ratings of Experiment 2, we again collected subjective grouping ratings for the vertically arranged Ts using the critical spacing of 1.7 degrees (see below) between the Vertical flankers and the target. Grouping strength was higher for Matched than for Unmatched stimuli (5.5 vs 3.5, t(4) = 2.902, p<0.05).

In Experiment 3B, the Vertical flankers were presented at four different horizontal positions: At the target position (8 degrees), the innermost Horizontal flanker position (6 degrees) and two positions in between the target and the innermost flanker (6.66 and 7.33 degrees). The Vertical flankers were always presented outside the critical spacing of crowding at each observer’s individual value (see below). Again, two blocks of 120 trials each were performed with each spacing condition.

In contrast to Experiment 2 where the individual critical spacing of the Vertical flankers was based on the orientation discrimination task from Experiment 1 (with four alternatives) potentially overestimating the critical spacing in the Yes/No task, we here determined the individual critical spacing by using the same Yes/No task as in the main Experiments 3A and 3B. Targets and Vertical flankers (without Horizontal flankers) at the five different distances to the target were presented. Psychometric functions (cumulative Gaussians) were fitted to the proportion correct data. The critical spacing for crowding was defined as the target-flanker distance at which performance reached a value of 90% correct responses. The distances between the target and the Vertical flankers for the seven observers were two times 1.4°, 1.6°, three times 1.7°, and 2.1° (center-to-center distance). Hence, on average the Vertical flankers ceased to crowd the target at distances larger than 1.7° or 0.21 times the eccentricity of the target. Note that this estimated critical spacing is smaller than in Experiment 1 (2.6°) what is presumably (at least partly) due to the different tasks used to establish the critical spacing (orientation discrimination with four alternatives and Yes/No task, respectively).

### Results

**Figure 7:**
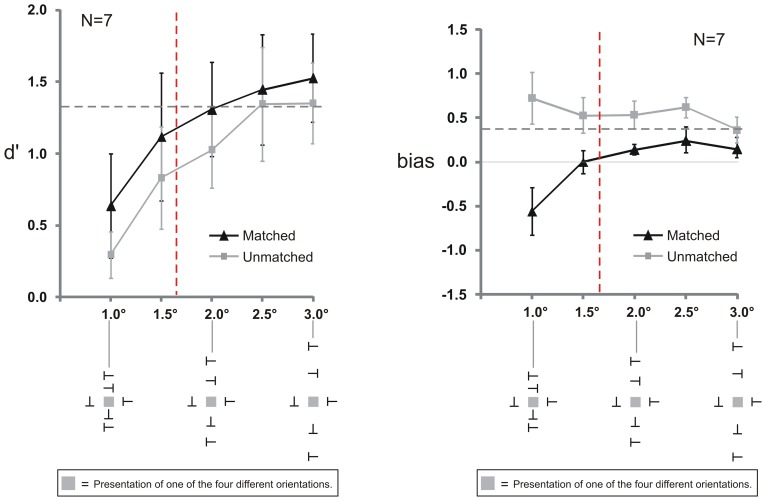
Results of Experiment 3A. Left panel: Sensitivity (d′) increased with larger spacing between the target and the Vertical flankers. There was a trend for higher sensitivity in the Matched compared to the Unmatched condition but no significant difference. The dashed horizontal line indicates d′ in the baseline condition where only Horizontal flankers were presented. The red dashed vertical line shows the calculated critical spacing of the Vertical flankers where performance reached 90% – Vertical flankers did not crowd the target at spacings larger than 1.7° (to the right of the red line). Right panel: Bias differences between the Matched and Unmatched condition were observed when the Vertical flankers were presented within the critical spacing (spacing smaller than 1.7°, to the left of the vertical red line) and when they were presented outside the critical spacing (spacing larger than 1.7°) up to and including 2.5°. Both panels: The stimulus icons depict a Grouped condition with upright Vertical flankers. In the Ungrouped condition, adjacent Vertical flankers had different orientations. Stimuli are not drawn to scale. The gray squares indicate the target position and were not presented in the experiment. Error bars indicate standard errors of the mean.

As we will see, Experiment 3A shows again that the grouping effect of the Matched conditions had no effect on sensitivity but did affect the biases. Experiment 3B shows that the bias strongly depended on the horizontal position of the Vertical flankers, arguing against a response bias and for a perceptual bias. Figure 7 shows the results of Experiment 3A. The data were analyzed with repeated measures two-way ANOVAs with the two factors Spacing (1.0°, 1.5°, 2.0°, 2.5°, and 3.0°) and Matching (Matched and Unmatched). Separate ANOVAs were conducted for the dependent variables sensitivity (d′) and bias.

Sensitivity (d′, left panel of Figure 7) increased with increasing Vertical flanker spacing with the smallest average d′ (0.46) at a spacing of 1.0° and the highest average d′ (1.44) at a spacing of 3.0° (main effect of Spacing: F(4, 24) = 4.48, p<0.01). This effect shows that at smaller spacing conditions Vertical flankers crowded the target (in addition to the crowding by the Horizontal flankers, d′ = 1.32), and that crowding by the Vertical flankers decreased with larger spacing. Despite the estimated critical spacing of 1.7° (indicated by the red dashed vertical lines in Figure 7), d′ seems to increase at spacings beyond 1.7°, potentially indicating additive crowding effects of the Vertical and Horizontal flankers beyond the critical spacing. However, contrasts revealed that this was not the case. There was a difference between d′ values at a spacing of 1° and the largest spacing – with the highest d′ values – at 3° (F(1,54) = 5.694, p<0.05), revealing additional crowding by the Vertical flankers at 1°. No such difference was found between d′ values at 1.5° and 3° (F(1,54) = 1.301, p = 0.259), indicating that there was no additional crowding by the Vertical flankers already at a spacing of 1.5°, and hence, no additive effects of the Vertical and Horizontal flankers beyond this spacing.

There was a trend for higher sensitivity in the Matched compared to the Unmatched condition, however, no main effect of Matching (F(1,6) = 5.05, p = 0.066) and no interaction was observed (F(4,24) = 0.279; p = 0.889). Grouping by the Matched Vertical flankers did not affect sensitivity.

Bias results are shown on the right panel of Figure 7. We found a main effect of Matching (F(1,6) = 10.043, p<0.05) and an interaction (F(4,24) = 5.78, p<0.05). Planned comparisons revealed that the Matched and Unmatched conditions differed at each spacing up to (and including) the second largest spacing of 2.5° (F(1,54) = 6.49, p<0.05). Next, to use a more conservative estimate of the critical spacing between the target and the Vertical flankers, we defined the critical spacing as the distance where performance reached 95% correct responses instead of 90%. Fitting a psychometric function to the average proportion correct data yielded a spacing of 1.95° at 95% correct responses. The spacing of 2.5° is still larger than this newly estimated critical spacing. There was no bias difference between the Matched and Unmatched condition at the largest spacing of 3.0° (F(1,54) = 2.14, p = 0.149).

There was no main effect of Spacing (F(4,24) = 2.283, p = 0.09), however, the *difference* between the Matched and Unmatched condition depended on the spacing (see significant interaction) showing that the effect of grouping on bias was greatest at close spacing but persisted for spacings beyond the critical spacing of crowding.

**Figure 8:**
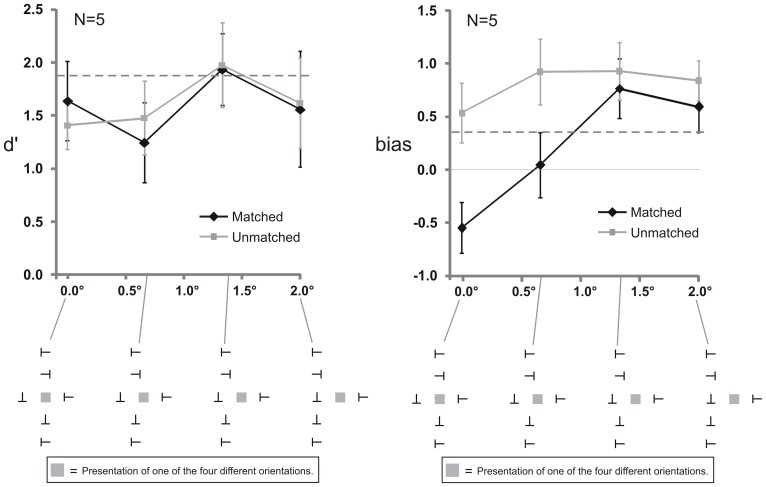
Results of Experiment 3B. Bias depends on alignment. The X-axis in both panels shows the horizontal distance of the Vertical flankers to the target where 0.0° indicates that the Vertical flankers were aligned with the target and 2.0° that they were aligned with the innermost flanker. Stimuli are depicted as presented to the right side of fixation. Left panel: There were no sensitivity (d′) differences between the Matched and Unmatched condition. Sensitivity did not differ between the four spacing conditions. The dashed horizontal line indicates d′ in the baseline condition were no Vertical flankers were presented. Right panel: Bias differences between the Matched and Unmatched condition were observed when the Vertical flankers were presented aligned with the target (0.0° horizontal distance) or close to the target (0.66°). At larger distances (1.33° and 2.0°), no difference between the Matched and Unmatched condition were observed. Both panels: Error bars indicate standard errors of the mean. The gray square indicates the target position and was not presented in the experiment. Stimuli are not drawn to scale.

Figure 8 shows the results of Experiment 3B. The data was analyzed with repeated measures two-way ANOVAs with the two factors Horizontal Spacing (horizontal distance of the Vertical flankers from the target: 0.0°, 0.66°, 1.33°, and 2.0°) and Matching (Matched and Unmatched). Separate ANOVAs were conducted for sensitivity (d′) and bias.

Sensitivity (left panel of Figure 8) did neither differ between the different Horizontal Spacing conditions (F(3,12) = 0.791, p = 0.522) nor between the Matched and Unmatched condition (F(1,4) = 0.028, p = 0.876), and no interaction was observed (F(3,12) = 0.768, p = 0.534).

The right panel of Figure 8 shows bias results. There was no main effect of Horizontal Spacing (F(3,18) = 3.026, p = 0.056). A main effect of Matching (F(1,6) = 10.973, p = 0.016) and an interaction (F(3,18) = 4.961, p = 0.011) were observed. Planned contrasts revealed bias differences between the Matched and Unmatched conditions when the horizontal spacing was 0.0° (F(1,28) = 7.132, p = 0.025) and when it was 0.66° (F(1,28) = 4.72, p = 0.0096) but not when the spacing was 1.33° (F(1,28) = 0.166, p = 0.687) and 2.0° (F(1,28) = 0.3707, p = 0.548).

These results argue against a response bias as the source of the bias from the Vertical flankers when they are all the same. Specifically, the salience of the several, identical Vertical flankers should not depend on the alignment and it must be this salience that triggers any response bias.

Note that in contrast to Experiment 2, the Vertical flankers presented outside the critical spacing in Experiment 3 were closer to the target (on average at a distance of 1.7°) than the Horizontal flankers (at 2.0°) for all observers except one. Hence, grouping by proximity was stronger between the target and the Vertical flankers than between the target and the Horizontal flankers. Due to the radial-tangential asymmetry of crowding, the closer Vertical flankers did not crowd the target while the farther Horizontal flankers did crowd the target.

## General Discussion

Crowding and grouping share a large number of characteristics. Here, we investigated whether effects of crowding and grouping on target perception are subject to the same spatial constraints. We have presented evidence suggesting that grouping like crowding can affect target appearance but that grouping acts over a greater spatial range than does crowding. The effect of grouping that we measured appears to operate through an assimilation of the target to the common identity of the grouping elements.

There are many reasons to see crowding and grouping as quite similar. For example, both depend strongly on the spacing and the similarity between items (Figure 1; e.g., law of proximity in grouping; [8], [18]–[21], but see [51]). A number of studies have shown that target-flanker grouping [24], [36]–[41] and flanker-flanker grouping [34]–[35] are crucial in crowding. In particular, it was suggested that crowding strength depends on how much the target groups with the flankers – the stronger the target-flanker grouping the stronger the crowding, and the more the target stands out from the flankers the weaker the crowding, e.g., [37].

We showed here that, contrary to the many similarities between crowding and grouping, they differ in regard to the critical spacing affecting target perception. Flankers outside the critical spacing of crowding had different effects when they had the same orientation, (and the same orientation as the target in Experiment 1), i.e. when they grouped, or different orientations (different from the target in Experiment 1), i.e. when they did not group. While in Experiment 1 performance was better when the Vertical flankers had the same orientation as the target, the results of Experiments 2 and 3 indicated that this was not due to increased sensitivity but due to a bias to report target present in the Matched condition and target absent in the Unmatched condition. To determine whether a response bias had caused this effect, we varied the horizontal position of the Vertical flankers in Experiment 3B. We found the bias differences between the Matched and Unmatched condition only when the Vertical flankers were aligned (or nearly aligned) with the target. When the Vertical flankers were aligned with the innermost Horizontal flanker or closer to the innermost Horizontal flanker than to the target, no difference between the Matched and Unmatched condition was observed. This clear spatial dependence of the effect argued against a simple response bias (i.e., observers reporting salient items as targets).

Most explanations of crowding, such as substitution or pooling, can explain biases introduced by flankers when the flankers are close to the target, i.e., when they are presented within the critical spacing. Flanker features substitute for or are pooled with target features biasing responses in favor of the flankers. However, we here showed that flankers outside the critical spacing still modulate target identification. This cannot be explained by these integration accounts. Flankers that do not fall within the critical spacing should neither be substituted nor pooled with target features, and, so should not modulate target perception. Moreover, complete substitution, i.e. wrongly reporting a remote item from the Vertical set instead of the target can be excluded as well, as again such location errors only occur when the target-flanker spacing is small enough for crowding to occur [14], see also [13], [52]. Therefore, it is unlikely that a response substitution introduced by the Vertical flankers underlies the present results (see also response bias control, Experiment 3B). The same argument holds for contour integration, which has been linked to crowding [53]. While contour integration between aligned Ts might be a candidate to explain the present results, the association field in contour integration is smaller and assumed to resemble the critical spacing for crowding [53], see also [54].

Taken together, standard crowding explanations cannot explain the present results because the Vertical flankers simply did not crowd the target when they were presented outside the critical spacing. Hence, the difference between Matched and Unmatched Vertical flankers in these conditions is not caused by crowding. Response bias was one candidate explanation, however, this explanation was ruled out by Experiment 3B.

We suggest instead that the observed effects are due to a change in the target’s appearance caused by grouping, where an element’s shape is assimilated to the common shape of the group. When the Vertical flankers had the same orientation as each other, we suggest that they made the target appear to be of the same orientation as the Matched (Grouped) flankers. This shape change produced better performance in the Grouped condition compared to the Ungrouped condition in Experiment 1 and a higher rate of reporting target present in the Matched compared to the Unmatched condition in Experiments 2 and 3 (whether or not the target was present).

**Figure 9:**
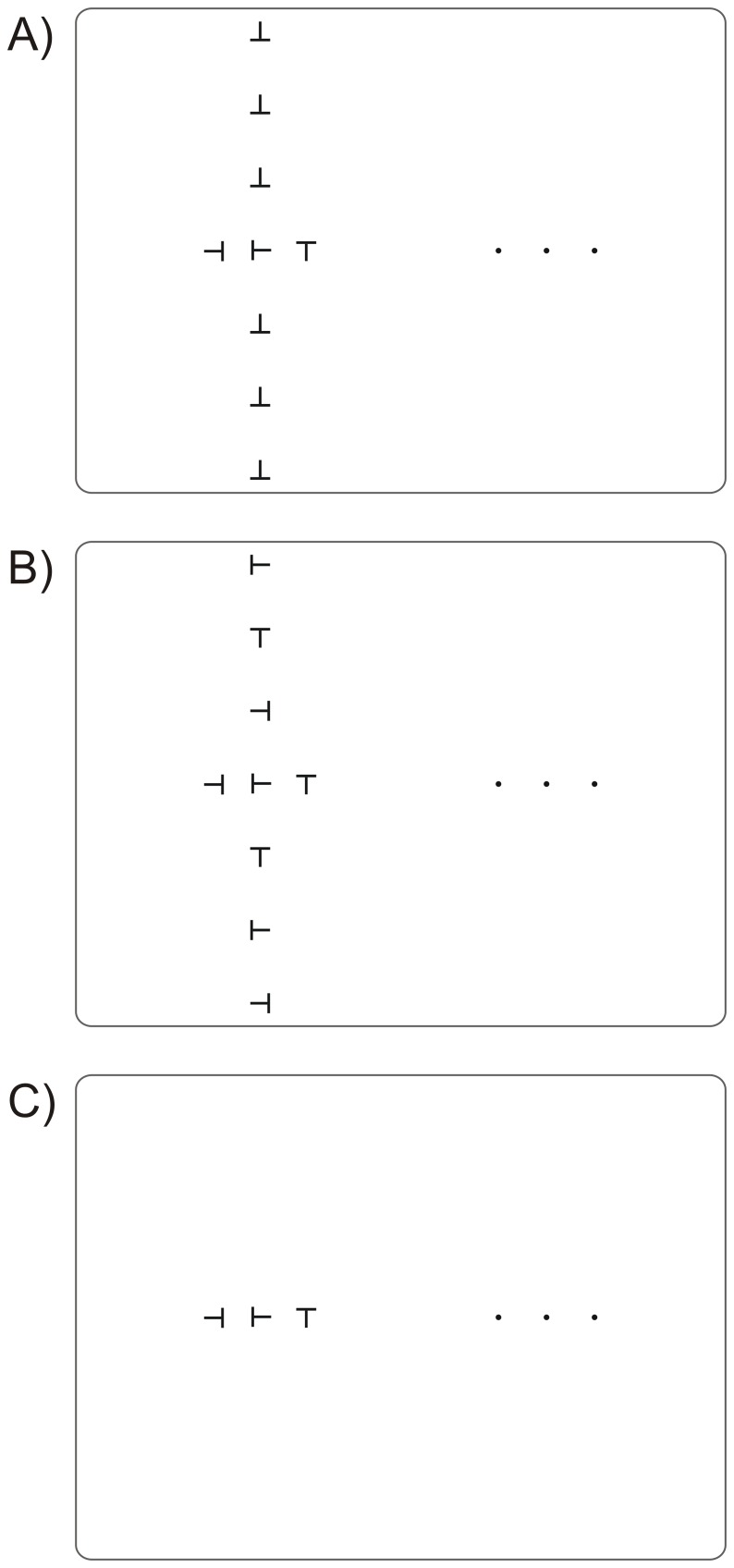
Demonstration of effects of grouping, screenshots of Movies S1 to S3. Please see Movies S1 to S3 and instructions in the Supporting Information section. A) Matched condition, B) Unmatched condition, C) Baseline.

Grouping is an ill-defined perceptual process that appears to work on at least two different levels. First, the elements of a group appear to belong together and second, elements in the group also look more alike. This change in the perceived appearance of the grouped elements is called assimilation, e.g., [44]–[45], see also [42], [55]–[58]. Seeing elements of a group as belonging together does not necessarily entail a change of their appearance. For example, when presenting a number of different objects arranged in a straight line, these objects may appear to belong together, i.e. to be grouped, but it does not make them all look the same. Here, as in other examples of shape assimilation in grouping, e.g., [42] the target visibility was weakened (in our case by crowding from the Horizontal flankers). With the target shape less well defined, we assume that it was then susceptible to the perceptual assimilation effect of grouping. While the change of target appearance we observed here may be related to a recently reported change of target appearance in crowding [50], our results show that perceptual assimilation by grouping operates over larger distances than the critical spacing in crowding. A demonstration of the perceptual nature of the assimilation effect of grouping may be seen in the movies in Figure 9. Based on our results, we suggest that although crowding and grouping share many characteristics and both may have effects on target appearance, differences between these two processes, such as the difference of their spatial extents shown here, will help to shed light on the underlying mechanisms of crowding and grouping alike.

## Supporting Information

Movie S1 **Matched condition.** There are three dots on the right for fixation. Try all three but choose one that produces some crowding, making the central target, a left-pointing T, difficult to identify. Click on the start button, at the lower left in Movie S1, to begin the cycling presentation of the target and flankers. In this first movie, the Vertical flankers are all aligned and upside-down, and here you may see the central target as also upside-down, or at least having a horizontal stroke at the bottom of the target. If you do, you are seeing the assimilation effect of grouping where the percept of the target is altered to be more like the aligned, identical, grouped flankers above and below.
(MOV)

Movie S2 **Unmatched condition.** In this movie, the Vertical flankers are randomly oriented. Check here if you see the target with the jumbled appearance common to crowded targets.
(MOV)

Movie S3 **Baseline.** Finally, in this movie, there are no Vertical flankers and the target may again appear jumbled. If the stimulus appears too jumbled in all the movies when fixating the outer dot, try fixating the middle or the inner dot.
(MOV)
